# Effect of Da-Cheng-Qi Decoction on the Repair of the Injured Enteric Nerve-Interstitial Cells of Cajal-Smooth Muscle Cells Network in Multiple Organ Dysfunction Syndrome

**DOI:** 10.1155/2014/596723

**Published:** 2014-11-13

**Authors:** Mu-Cang Liu, Ming-Zheng Xie, Bin Ma, Qing-Hui Qi

**Affiliations:** Department of General Surgery, The First Affiliated Hospital of Dalian Medical University, Liaoning 116011, China

## Abstract

Wistar rats were randomly divided into control group, multiple organ dysfunction syndrome (MODS) group, and Da-Cheng-Qi decoction (DCQD) group. The network of enteric nerves-interstitial cells of Cajal- (ICC-) smooth muscle cells (SMC) in small intestine was observed using confocal laser scanning microscopy and transmission electron microscopy. The results showed that the numbers of cholinergic/nitriergic nerves, and the deep muscular plexus of ICC (ICC-DMP) and connexin43 (Cx43) in small intestine with MODS were significantly decreased. The network integrity of enteric nerves-ICC-SMC was disrupted. The ultrastructures of ICC-DMP, enteric nerves, and SMC were severely damaged. After treatment with DCQD, the damages were repaired and the network integrity of enteric nerves ICC-SMC was significantly recovered. In conclusion, the pathogenesis of gastrointestinal motility dysfunction in MODS in part may be due to the damages to enteric nerves-ICC-SMC network and gap junctions. The therapeutic mechanism of DCQD in part may be that it could repair the damages and maintain the integrity of enteric nerves ICC-SMC network.

## 1. Introduction

Multiple organ dysfunction syndrome (MODS) is the main cause of the death of patients with abdominal surgical diseases, which are characterized by hypermetabolizability, hypercirculation, immoderate, and out of controlled inflammatory response and organ dysfunction [1–3]. Its etiology is various, its pathogenesis is elusive, and its mortality is very high.

Studies have found that gastrointestinal (GI) tract is the key organ to originate and trigger systemic inflammation response syndrome (SIRS) and MODS [4, 5]. The condition of GI function is thought to be the criterion of evaluating the prognosis of critical patients [6, 7]. In the treatment of GI dysfunction, the recovery of GI motility is very important. Improving the recovery of GI motility could effectively prevent MODS from deteriorating to multiple organ failure. So, it is necessary to investigate the pathogenesis of GI motility dysfunction and look for effective treatments for MODS [8, 9].

Interstitial cells of Cajal (ICC) show a highly branched morphology and form unique network in GI tract. ICC serve as electrical pacemakers, active propagation pathways for slow waves, and mediators of enteric motor neurotransmission. They play an important role in generating and regulating of GI motility. Enteric nerves system (ENS), ICC, and smooth muscle cells (SMC) connect to form a network structure, which is the basic functional unit of GI motility [10]. Gap junctions connect ICC to ICC, ICC to SMC, and SMC to SMC [11–14]. Connexin (Cx) is the basic structural and functional proteins that form gap junction channel. Studies have shown that, in GI motility dysfunction diseases, there is abnormality or decrease in the number of ICC [15, 16], or decrease in neurotransmission of ENS-ICC-SMC network [17–19], or abnormal expression of Cx [20].

In clinical practice, Chinese researchers have found that purgative therapy have a good effect on prevention and treatment of MODS. Da-Cheng-Qi decoction (DCQD) is compound preparation, composed of Rhubarb, Mirabilite, Fructus Aurantii Immaturus, and Mangnolia officinalis. DCQD has been used as purgative for nearly two thousand years in China. Researchers have found it was very effective in promoting the recovery of GI motility [21–23]. However, the mechanism is not fully understood.

In this study, the rat models with MODS induced by bacterial peritonitis were investigated, the morphological changes of ENS-ICC-SMC network in rats were observed, and the therapeutic effect of DCQD was investigated.

## 2. Materials and Methods

### 2.1. Materials

One hundred adult Wistar rats (200 g–250 g), half male and half female, were provided by experimental animal center of Dalian Medical University.* Escherichia coli* (*E. coli*) strain was provided by clinical laboratory of the First Affiliated Hospital of Dalian Medical University. Da-Cheng-Qi granules (batch number: 20130108) were provided by pharmaceutical factory of Tianjin Nankai Hospital, each dose drug was made of crude drug of 12 g of Rhubarb, 6 g of Mirabilite, 9 g of Fructus Aurantii Immaturus, and 9 g of Magnolia officinalis. Each dose drug was added into 36 mL distilled water and dissolved into liquid containing 100% crude drugs (1 g/mL).

### 2.2. Animal Grouping and Model Preparation

According to the random number table method, rats were grouped as follows: control group, twenty rats, each rat was intraperitoneally infused with 1 mL saline; MODS group, forty rats, each rat was intraperitoneally infused with 1 mL* E. coli* suspensions to establish the rat model of MODS; the DCQD group, forty rats, each rat was intraperitoneally infused with 1 mL* E. coli* suspensions to establish the rat model of MODS. In the same day, each rat was given DCQD by gavage, two times/days, each time 1 mL/100 g.

The study protocol was approved by the ethics committee of the First Affiliated Hospital of Dalian Medical University.

Diagnostic criteria of rat model of MODS [24]: respiratory distress and respiratory frequency ⩾ two times contrast value; tachycardia and heart rate ⩾ two times contrast value; abdominal bloating and bowel sounds are weakened or disappeared; blood bilirubin or glutamic-pyruvic transaminase ⩾ two times contrast value.

### 2.3. The Deep Muscular Plexus of ICC (ICC-DMP) and Cholinergic/Nitrergic Nerves-ICC Network in Intestinal Tissues Using Confocal Laser Scanning Microscopy

Forty-eight hours, respectively, after operation, the rats were killed with anesthesia. Ten-centimeter small intestine near pylorus was removed, cut into 1-2 cm fragments, fixed on zamboni liquid, and placed in refrigerator, 4°C, overnight. Dissected with anatomic microscope, the mucosa and submucosa as one layer were striped and complete intestinal muscularis was retained.

In order to investigate ICC-DMP and cholinergic nerves-ICC network, double stained with c-Kit and vesicular acetylcholine transporter (VAChT) immunohistochemistry according to Nemeth's method [25]: after finishing the whole-mount preparation, (1) the specimens were incubated in 0.5% Triton-X in 0.05 mol/L Tris-HCl buffer (pH 7.6) solution at 37°C for 4 hours; (2) after rinsing twice in PBS, the specimens were incubated 1 hour at room temperature in 1%BSA to prevent a specific linking; (3) after rinsing twice in PBS, the tissues were incubated 48 hours at 4°C in goat anti-rat VAChT polyclonal antibody (Santa Cruz, Biotech, USA); (4) the tissue samples were again rinsed twice in PBS and incubated with Texas Red-labeled donkey anti-goat IgG antibody (Santa Cruz, Biotech, USA) for 2 hours at room temperature; (5) after rinsing twice in PBS, the tissues were incubated 48 hours at 4°C in rabbit anti-rat c-kit polyclonal antibody (c-19, Santa cruz, Biotech, USA); (6) the whole tissue specimens were rinsed twice and incubated 2 hours at 4°C away from light in FITC-mouse anti-rabbit IgG antibody (Biotech, USA); (7) the tissue specimens were embedded into fluorescence mounting medium and investigated with confocal laser scanning microscopy.

In order to investigate ICC-DMP and nitrergic nerves-ICC network, it was double stained with C-Kit and neuronal nitric oxide synthase (nNOS) immunohistochemistry according to Nemeth's method [25]. It was the same way as above, but we added goat anti-rat nNOS polyclonal antibody (Santa Cruz, Biotech, USA) instead of VAChT polyclonal antibody in step (3).

Control group added antibody diluent only instead of antibody in steps (3) and (5).

Specimens were observed using a TCS-SP2 laser scanning confocal microscope (Leica, Germany) with immersion objectives (×40 numerical aperture 1.3). Tissue specimens were excited using a krypton/argon laser with excitation and barrier filters set for individual fluorophores according to their specific excitation-emission spectra (FITC = 494 nm and Texas Red = 595 nm). The emitted light was detected by a photomultiplier tube and converted via an analog-to-digital converter into a digital pixelated image. The detection pinhole was set for use with different objectives accordingly. C-Kit positive fluorescence was green and VAChT positive fluorescence was red. Three specimens were observed in each group and three high magnification visions were observed randomly in each specimen. Three-dimensional images reconstruction and fluorescence quantitative were performed by Leica confocal software.

### 2.4. Cx43 in Intestinal Tissues Using Light Microscopy

The same specimens of small intestine were cut into 1 cm long fragments, fixed in formaldehyde, and placed in refrigerator, 4°C, overnight. Thereafter, 2 mm long tissue was removed, dehydrated using graded ethanol, dewaxed using dimethyl benzene, and cut to serial sections, 5 *μ*m thick, using Leitz1512 slicer. Tissue biopsies were dried for 4 hours at 60°C in oven. (1) they were dewaxed by dimethyl benzene and dehydrated using graded ethanol; (2) they were put in hydrogen peroxide methanol to block endogenous peroxidase; (3) they were put in 0.1 mol/L citrate buffer solution (PH6.0), boiling for 20 minutes, for antigen repair; (4) after rinsing three times in PBS, they were incubated with normal goat serum for 20 minutes at room temperature; (5) after removing excess serum, they were incubated with mouse anti-rat connexin43 monoclonal antibody (CXN-6, Santa Cruz, Biotech, USA) (diluted to 1 : 200) and placed in refrigerator, 4°C, overnight; (6) after rinsing three times in PBS, they were incubated with biotin-labeled sheep anti-mouse IgM antibody for 30 minutes at room temperature; (7) after rinsing three times in PBS, they were incubated with ABC kit for 30 minutes at room temperature; (8) they were colored by DAB for 5–8 minutes; (9) they were stained with hematoxylin for 30 seconds; (10) they were blued with water for 30 minutes; (11) they dehydrated using graded ethanol; (12) vitrification was by dimethyl benzene; (13) they were mounted using neutral gum; (14) in control group, antibody diluent was added only instead of antibody in steps (5).

Tissue biopsies were observed using Nikon light microscope. Six specimens in each group and five high magnification visions in each specimen were observed randomly. Images were analyzed according to Nemeth's method [25].

### 2.5. Cx43 in Small Intestinal Muscle Strips Using Transmission Electron Microscopy

In the same specimens of small intestine, 10 cm small intestine near pylorus was removed and cut into 1 mm × 2 mm fragments. (1) Tissues were laid flat on filter paper, fixed in 4% glutaraldehyde, and rinsed in 0.1% phosphate buffer. Thereafter, they were fixed in 1% osmium tetroxide and rinsed in 0.1% phosphate buffer, (2) they were dehydrated using graded ethanol; (3) after fixed in osmic acid, they were dehydrated using graded ethanol, substituted using epoxypropane, and embedded using Epon812; (4) in semiultrathin sections, 1~2 *μ*m, they stained with 2% toluidine blue and observed using light microscope. Distinguishing the location of mucosa, submucosa, circular muscularis, and longitudinal muscularis, the myenteric plexus and deep nerve plexus were made into ultrathin section; (5) ultrathin sections (50–70 nm thick) were put on copper reseau with 200 or 400 holes. (6) after staining with uranyl acetate, stained with citrate; (7) tissue biopsies were observed using JEM-2000EX transmission electron microscope. The images collection and statistical analysis were completed.

### 2.6. Statistical Analysis

The data was analyzed using a commercial software package (SPSS 13.0). Data of ICC-DMP and ENS-ICC network in intestinal tissues were expressed as mean ± standard deviation,* t*-test was applied for difference comparison among each group, and *P* < 0.05 denoted the difference possessing statistical significance. Data of Cx43 in intestinal tissues using light microscopy was analyzed by rank sum test, and *P* < 0.05 denoted the difference possessing statistical significance.

## 3. Results

Forty-eight hours later, in control group, all 20 rats survived; in MODS group, twenty-four hours later, 30 rats reached the criterion of MODS and, forty-eight hours later, 21 rats died. In DCQD group, twenty-four hours later, 29 rats reached the criterion of MODS and, forty-eight hours later, 12 rats died. There was no difference in occurrence time and incidence of MODS between MODS group and DCQD group. But, we found that the rats in MODS group ate less than DCQD group. The mortality in DCQD group was lower than that in MODS group (41.4% versus 70.0%, Chi-square test, *P* < 0.05).

### 3.1. GI Gross Specimen

**Figure 1:**
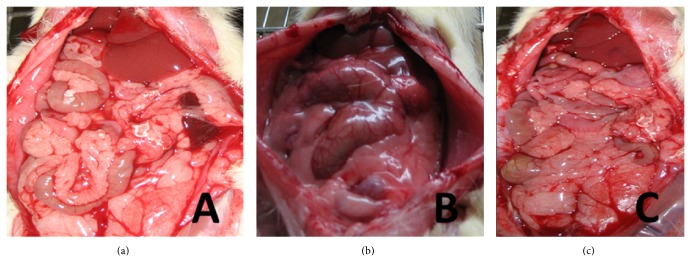
GI gross specimen. (a) It shows GI gross specimen of control group; (b) it shows GI gross specimen of MODS group; (c) it shows GI gross specimen of DCQD group.

Control group (Figure 1(a)) shows the GI tract was normal in appearance: pink, no adhesion, and regular bowel movements could be seen.

MODS group (Figure 1(b)) shows the GI tract was extremely distended and swelling, most of the bowel was dark, and there was bloody ascites in some rats. Moreover, we found that there were dark bloody liquid in lumen, bleeding spots and necrosis on intestinal mucosa, bleeding spots on great omentum, and a lot of exudation in abdominal cavity. We also found that bowel movements disappeared.

DCQD group (Figure 1(c)) shows the GI tract was red, lightly distended, and swelling. Though there was a small amount of pale yellow liquid in lumen and a small amount of exudation in abdominal cavity, there were no bloody ascites. Moreover, bowel movements were nearly normal.

### 3.2. ICC-DMP Using Confocal Microscopy

**Figure 2:**
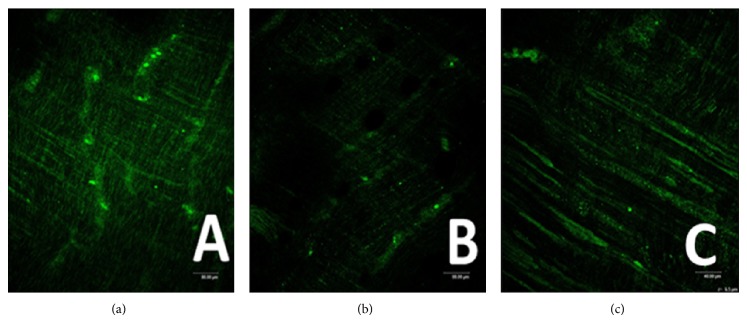
ICC-DMP using confocal microscopy. (a) Control group shows ICC-DMP connected to each other and formed network (Ruler: 80 *μ*m); (b) MODS group shows there was an obvious decrease in numbers of ICC and synapses. Moreover, network integrity was damaged. IOD was also markedly decreased (ruler: 80 *μ*m); (c) DCQD group shows ICC and synapses of ICC were more than MODS group, maintaining net structure (ruler: 40 *μ*m).

Control group (Figure 2(a)) shows ICC-DMP were spindle-shaped cells with 2-3 synapses, these cells connected to each other by synapses, and they formed network structure.

**Figure 3:**
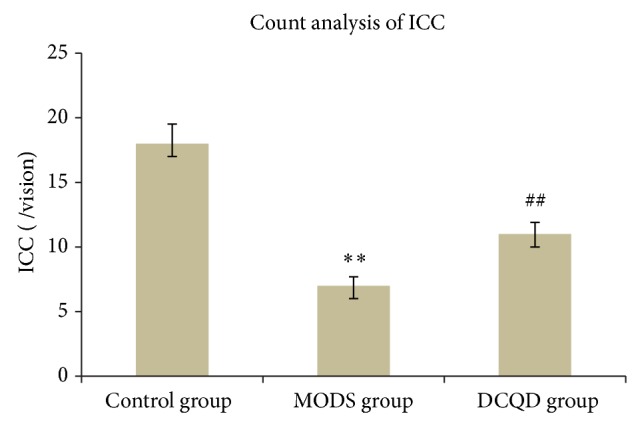
Count analysis of ICC. Three specimens in each group and three high magnification visions in each specimen were observed randomly. Compared with control group, *n* = 9, ^**^ *t* = 8.295, *P* < 0.01; compared with MODS group, *n* = 9, ^##^ *t* = 4.041, *P* < 0.01.

**Figure 4:**
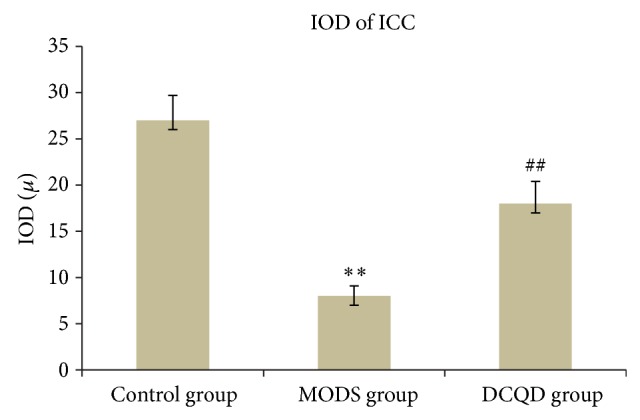
IOD of ICC. Three specimens in each group and three high magnification visions in each specimen were observed randomly. Compared with control group, *n* = 9, ^**^ *t* = 6.536, *P* < 0.01; compared with MODS group, *n* = 9, ^##^ *t* = 3.917, *P* < 0.01.

MODS group (Figures 2(b), 3, and 4) shows that not only there was an obvious decrease in numbers of ICC and synapses, but network integrity was damaged. Integrated optical density (IOD) of ICC was also markedly decreased.

DCQD group (Figures 2(c), 3, and 4) shows the numbers of ICC and synapses of ICC were more than MODS group and ICC formed network structure. IOD of ICC was also markedly elevated and compared with MODS group.

### 3.3. Cholinergic/Nitrergic Nerves-ICC Network Using Confocal Microscopy

**Figure 5:**
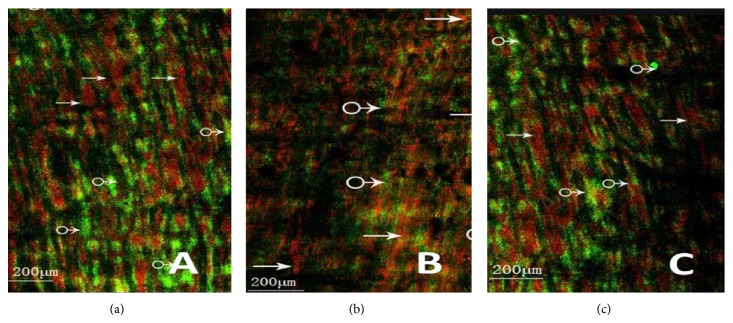
Cholinergic nerves-ICC network using confocal microscopy. (a) It shows cholinergic nerves-ICC network of control group (→: nerve fiber, ♂: ICC (ruler: 200 *μ*m); (b) it shows cholinergic nerves-ICC network of MODS group (→: nerve fiber, ♂: ICC). Nerve fibers and ICC were significantly reduced and the nerves-ICC network integrity was damaged (ruler: 200 *μ*m); (c) it shows cholinergic nerves-ICC network of DCQD group (→: nerve fiber, ♂: ICC). Nerve fibers and neuronal connections were more than MODS group, maintaining net structure (ruler: 200 *μ*m).

**Figure 6:**
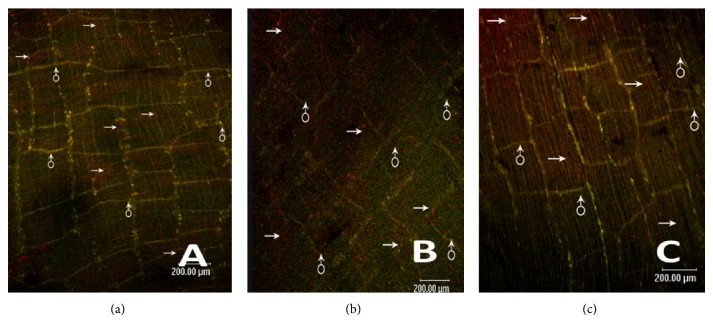
Nitrergic nerves-ICC network using confocal microscopy. (a) It shows nitrergic nerves-ICC network of control group (→: nerve fiber, ♂: ICC) (ruler: 200 *μ*m); (b) it shows nitrergic nerves-ICC network of MODS group (→: nerve fiber, ♂: ICC). Nerve fibers and ICC were significantly reduced and the nerves-ICC network integrity was damaged (ruler: 200 *μ*m); (c) it shows nitrergic nerves-ICC network of DCQD group (→: nerve fiber, ♂: ICC). Nerve fibers and neuronal connections were more than MODS group, maintaining net structure (ruler: 200 *μ*m).

Control group (Figures 5(a) and 6(a)) shows the dense c-Kit-positive cellular network (ICC-DMP), located between the longitudinal and circular muscularis and at the innermost part of the circular muscularis, and intermingled with the myenteric plexus. Between the circular muscle fibers, there was abundance of VAChT/nNOS-positive nerve fibers and c-Kit-positive cellular network run parallel with the muscle fibers. We also found that the VAChT/nNOS-positive myenteric plexus was surrounded by a reticular network of c-Kit-positive ICC.

**Figure 7:**
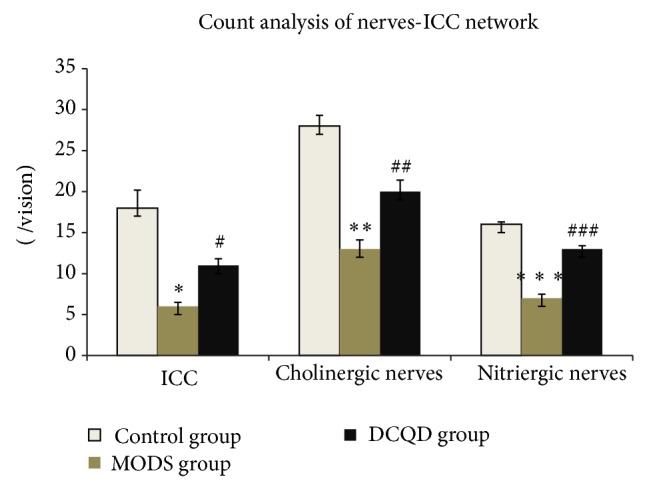
Count analysis of ICC, cholinergic nerve fibers, and nitrergic nerve fibers. Three specimens in each group and three high magnification visions in each specimen were observed randomly. Compared with control group, count analysis of ICC, *n* = 9, ^*^ *t* = 9.508, *P* < 0.01; compared with control group, count analysis of cholinergic nerve fibers, *n* = 9, ^**^ *t* = 8.793, *P* < 0.01; compared with control group, count analysis of nitrergic nerve fibers, *n* = 9, ^***^ *t* = 15.041, *P* < 0.01; compared with MODS group, count analysis of ICC, *n* = 9, ^#^ *t* = 5.598, *P* < 0.01; compared with MODS group, count analysis of cholinergic nerve fibers, *n* = 9, ^##^ *t* = 3.670, *P* < 0.01; compared with MODS group, count analysis of nitrergic nerve fibers, *n* = 9, ^###^ *t* = 9.500, *P* < 0.01.

**Figure 8:**
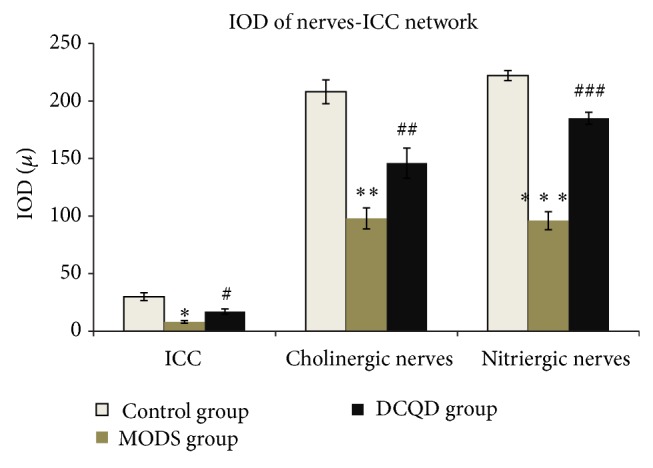
IOD of ICC, cholinergic nerve fibers, and nitrergic nerve fibers. Compared with control group, IOD of ICC, *n* = 9, ^**^ *t* = 6.293, *P* < 0.01; compared with control group, IOD of cholinergic nerve fibers, *n* = 9, ^**^ *t* = 8.032, *P* < 0.01; compared with control group, IOD of nitrergic nerve fibers, *n* = 9, ^***^ *t* = 13.946, *P* < 0.01; compared with MODS group, IOD of ICC, *n* = 9, ^#^ *t* = 3.851, *P* < 0.01; compared with MODS group, IOD of cholinergic nerve fibers, *n* = 9, ^##^ *t* = 2.999, *P* < 0.01; compared with MODS group, IOD of nitrergic nerve fibers, *n* = 9, ^###^ *t* = 9.349, *P* < 0.01.

MODS group (Figures 5(b), 6(b), 7, and 8) shows ICC and cholinergic/nitrergic nerve fibers were significantly reduced, the connections between nerves and ICC were reduced or disappeared, and cholinergic/nitrergic nerves-ICC network integrity was damaged significantly. IOD of ICC and nerve fibers was markedly decreased.

DCQD group (Figures 5(c), 6(c), 7, and 8) shows ICC, cholinergic/nitrergic nerve fibers, and neuronal connections were more than MODS group and formed network structure. IOD of ICC and nerve fibers was also markedly elevated, compared with MODS group.

### 3.4. Ultrastructures of ICC Using Conventional Electron Microscopy

**Figure 9:**
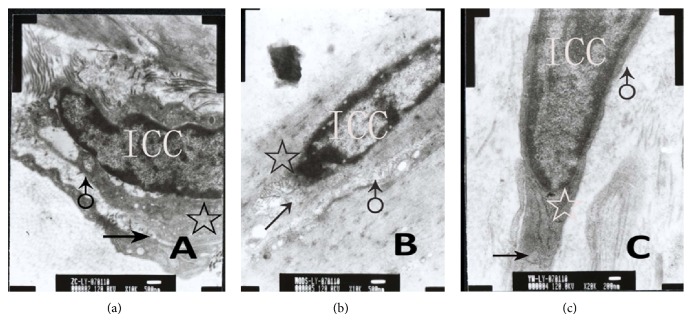
Ultrastructures of ICC-DMP using conventional electron microscopy. (a) Control group shows there were abundant mitochondria (☆), smooth endoplasmic reticulum, well-developed Golgi apparatus (↑), and intact basal membrane (♂) (ruler: 500 nm); (b) MODS group shows nucleus shriveled and the number of organelles decreased significantly. Also, mitochondria (☆) were distorting and swelling, and Golgi apparatus (↑) was damaged. Moreover, basal membrane (♂) was incomplete (ruler: 500 nm); (c) DCQD group shows nucleus was normal. Also, mitochondria (☆) and Golgi apparatus (↑) were more than MODS group. Only a few mitochondria were swelling and a few endoplasmic reticula were dilated. Moreover, basal membrane (♂) was almost complete (ruler: 200 nm).

Control group (Figure 9(a)) shows ICC were shuttle type, nucleus was big, and oval and chromatins structure was clear. There were abundant mitochondria, smooth endoplasmic reticulum, ribosomes, and few of rough endoplasmic reticulum in ICC, and Golgi apparatus were small. There was intact basal membrane.

MODS group (Figure 9(b)) shows nucleus shriveled, and the number of organelles was decreased significantly. We also found mitochondria were distorting and swelling, and endoplasmic reticulum was dilated. Moreover, we found the basal membrane was incomplete.

DCQD group (Figure 9(c)) shows nucleus was normal and the number of organelles was more than MODS group. Only a few of mitochondria were swelling and a few of endoplasmic reticula were dilated. Moreover, we found the basal membrane was almost complete.

### 3.5. Cx43 in Intestinal Tissues Using Light Microscopy

**Figure 10:**
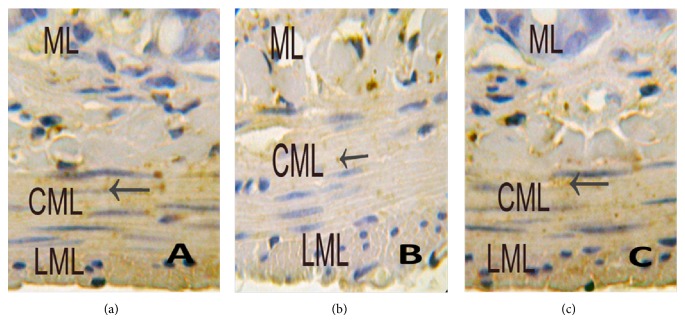
Immunohistochemistry of Cx43 using light microscopy. (a) Control group shows immunoreactive products of Cx43 (←) were tan and mainly located evenly in circular muscularis, little in longitudinal muscularis, and none in mucous layer (at magnification of 400 times); (b) MODS group shows immunoreactive products of Cx43 (←) were tan and mainly located in circular muscularis, less than control group, none in longitudinal muscularis and mucous layer (at magnification of 400 times); (c) DCQD group shows immunoreactive products of Cx43 (←) were tan and mainly located evenly in circular muscularis, and there was no significant difference, compared with control group (at magnification of 400 times).

Control group (Figure 10(a)) shows immunoreactive products of Cx43 were tan and mainly distributed in the cytomembrane and cytoplasm. In small intestinal muscularis, Cx43 was mainly located in circular muscularis and gaps between muscularis, especially intensively and evenly in outermost layer of circular muscularis and in the layer close to internal surface of mucosa, with little or no distribution in longitudinal muscularis.

MODS group (Figure 10(b)) shows immunoreactive products of Cx43 were mainly distributed in the cytomembrane and cytoplasm and were less than control group (*P* < 0.01). Moreover, we found that there was little or no distribution of Cx43 in small intestinal muscularis.

DCQD group (Figure 10(c)) shows immunoreactive products of Cx43 were more than MODS group (*P* < 0.01). Moreover, there was no significant difference, compared with control group.

According to Nemeth's [26] statistical criteria, the positive staining in cytomembrane and cytoplasm was tan. Immunoreactive products of Cx43 were divided into four grades by color density: (−) no staining (negative); (±) mild staining (weakly positive); (+) moderate staining (positive); (++) rich staining (strong positive) (see Table 1).

Six specimens in each group and five high magnification visions in each specimen were observed randomly. There was difference between control group, MODS group, and DCQD group using Kruskal Wallis test, *H* = 44.537, *P* < 0.05; there was difference between control group and MODS group using Mann-Whitney test, *H* = 67.500, *P* < 0.05; there was difference between MODS group and DCQD group using Mann-Whitney test, *H* = 118.500, *P* < 0.05; there was no difference between control group and DCQD group using Mann-Whitney test, *H* = 393.500, *P* > 0.05.

### 3.6. Cx43 Using Transmission Electron Microscopy

**Figure 11:**
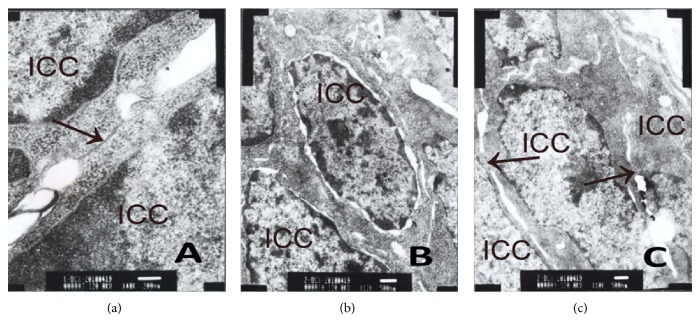
Cx43 between ICC and ICC using transmission electron microscopy. (a) Control group shows gap junction (↑) between ICC and ICC (ruler: 200 nm); (b) MODS group shows no gap junction between ICC and IC and ICC membrane was not clear. Moreover, chromatin condensation and aggregation could be seen (ruler: 500 nm); (c) DCQD group shows gap junction (↑) with intact structure between ICC and ICC. ICC nucleolus and cell membrane were clear. NO chromatin condensation and aggregation (ruler: 500 nm).

**Figure 12:**
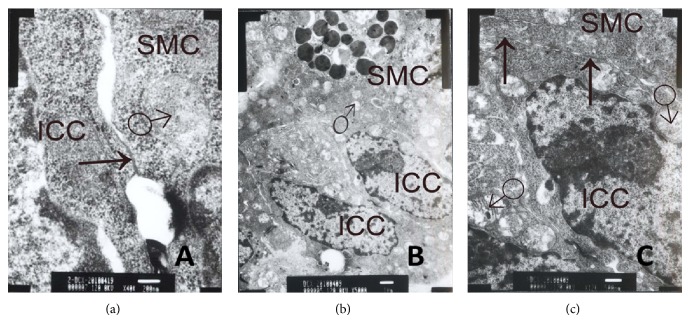
Cx43 between ICC and SMC using transmission electron microscopy. (a) Control group shows gap junction (↑) between ICC and SMC. Mitochondria (♂) (ruler: 200 nm); (b) MODS group shows no gap junction between ICC and SMC. Many swelling and damaged mitochondria (♂) in cell (ruler: 1 *μ*m); (c)DCQD group shows gap junction (↑) with intact structure between ICC and SMC. Many mitochondria (♂), and though some were swelling, there were no ruptured mitochondria (ruler: 500 nm).

**Figure 13:**
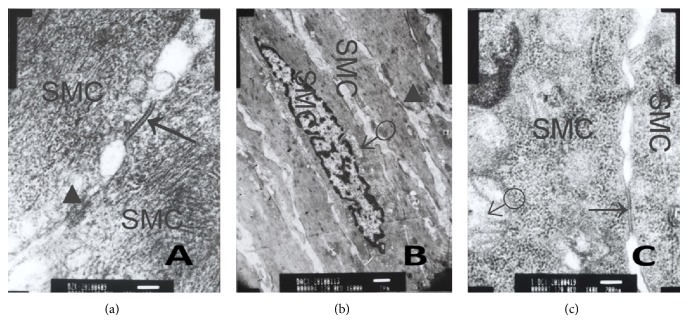
Cx43 between SMC and SMC using transmission electron microscopy. (a) Control group shows gap junction (↑) between SMC and SMC. Desmosomes junction (▲) between SMC and SMC. The distance was about 25–30 nm. There was also middle dense band or middle line in desmosomes junction and dense spot about 10 mm thick on the inside of plasma membrane (ruler: 100 nm); (b) MODS group shows no gap junction between SMC and SMC, and chromatin condensation and aggregation could be seen in SMC. Some mitochondria (♂) were swelling. There was desmosomes junction (▲) between SMC and SMC, dense spots of one desmosomes junction were asymmetric, and no middle line (ruler: 1 *μ*m); (c) DCQD group, gap junction (↑) with intact structure between SMC and SMC. There were many mitochondria (♂), and though some were swelling, there was no ruptured mitochondria. Moreover, cell membrane was clear (ruler: 1 *μ*m).

Control group (Figures 11(a), 12(a), and 13(a)) shows there were gap junctions between ICC and ICC, ICC and SMC, SMC, and SMC. The distance was about 2 nm. Many cylindrical combinations could be seen in gaps of ICC. We also found there were desmosomes junctions between ICC and ICC, ICC and SMC, and SMC and SMC, and the distance was about 25–30 nm. There was middle dense band or middle line in desmosomes junctions and dense spot about 10 mm thick on the inside of plasma membrane. ENS-ICC-SMC network was integrated.

MODS group (Figures 11(b), 12(b), and 13(b)) shows there were no gap junctions between ICC and ICC, ICC and SMC, SMC and SMC, and the distance obviously widened. We also found chromatin condensation and aggregation. Moreover, mitochondria were obviously swelling, some were ruptured, and cell membranes of ICC were oblique. ENS-ICC-SMC network was damaged significantly.

DCQD group (Figures 11(c), 12(c), and 13(c)) shows there were gap junctions between ICC and ICC, ICC and SMC, and SMC and SMC, and the distance was about 2 nm. Also, some cylindrical combination could be seen in gaps of ICC. Moreover, there were more mitochondria in ICC than MODS group, and cell membranes of ICC were clear. Though some mitochondria were swelling, nearly no mitochondria were ruptured, and there was no chromatin aggregation. ENS-ICC-SMC network was integrated.

## 4. Comment

MODS is an extremely complex systemic response process with many causes. It is the most serious complication after trauma and infection and its mortality is very high. Currently, the leading view is that MODS develops from SIRS and compensatory anti-inflammatory response syndrome.

Studies have shown that gut is the key organ to originate and trigger SIRS and MODS [4, 5]. MODS may cause gut ischemia and anoxia, thus resulting in increase in gut permeability. Increased gut permeability is conducive to the translocation of bacteria and endotoxin that lead to the activation of the immune inflammatory system, which contribute to SIRS/MODS [27–29]. MODS may cause GI peristalsis weakened or disappeared, so the digestive and absorption processes are affected and the bacteria in gut are overgrown. Then they produce large volumes of gas and the pressure in gastrointestinal tract will be increased. If the condition is further exacerbated, it will lead to toxic enteroparalysis which contribute to SIRS/MODS. So, improving the recovery of GI motility could effectively prevent MODS from deteriorating to multiple organ failure [8, 9]. But the pathogenesis of GI motility dysfunction in MODS is not fully understood. DCQD is compound preparation, composed of Rhubarb, Mirabilite, Fructus Aurantii Immaturus, and Magnolia officinalis. DCQD has been in use as purgative for nearly two thousand years in China. Researchers have found it is very effective in promoting the recovery of GI motility [21–23], However, the mechanism is not fully understood. In this study, we try to investigate the pathogenesis of GI motility dysfunction in MODS and therapeutic mechanism of DCQD.

In traditional Chinese medicine, for adults, the common dosage of crude drug of DCQD is 36–72 g. According to body surface area, we made dosage conversions between human and experimental rats, and for experimental rats, the dosage of crude drug of DCQD should be 0.38 g/100 g–0.76 g/100 g. But, in the preliminary experiment, we found that the best therapeutic dosage of DCQD to rats is 2 g/100 g (two times/days, each time 1 g/100 g), and we thought that rats may have stronger tolerance to DCQD than human. So, we chose the dosage of 2 g/100 g.

ICC serve as the pacemaker cells of the GI tract and mediators of enteric motor neurotransmission. They play an important role in generating and regulating GI motility. ICC have been grouped according to either their localization in the muscle layers, their basic morphology (stellate and bipolar), or their primary function [30]. ICC of the myenteric plexus (ICC-MY) occur around the circumference of the myenteric plexus in the space between the circular and longitudinal muscularis. ICC-MY serve as electrical pacemakers, active propagation pathways for slow waves; intramuscular interstitial cells of Cajal (ICC-IM) are the cell types found in the circular and longitudinal muscularis, which connect to swollen gastrointestinal motor nerve endings and are located surrounding muscle fibers in parallel. ICC-IM serve as mediators of enteric neurotransmission; ICC-DMP are the cell types found in the deep muscular plexus in the space between the inner layers of circular muscle layers and the outer layers of circular muscle layers and serve as mediators of enteric neurotransmission; ICC of the submucosa (ICC-SM) are the cell types found in the submucosa and are now considered to serve as pacemakers.

Studies have shown that there are synaptic-like contacts that connect ICC to enteric nerves [26] and gap junctions that connect ICC to SMC [31]. ENS, ICC, and SMC connect to form a network structure, which is the basic functional unit of GI motility [10, 29, 32–35]. ICC-DMP are the primary target cells of nerve fibers and express many neurotransmitter receptors, for example, muscarine receptors (M2 and M3), somatostatin 2A receptors, neurokinin receptors NK1 and NK3, vasoactive intestinal peptide (VIP-R), and so forth [36]. These indicate ICC serve as mediators of neural signal transduction to SMC. Studies have shown that VAChT, P substance (SP), nNOS, and vasoactive intestinal peptide (VIP) positive nerve fibers connect closely to ICC, and these provide a morphological basis for ICC serving as mediators of neural signal transduction [37–39]. Studies have shown that, in some GI motility disorders diseases, there is abnormality or decrease in number of ICC [15, 16] or decrease of neurotransmission of ENS-ICC-SMC network [17–19].

Studies have shown that, the signal transduction between ICC and ICC, ICC and SMC, and SMC and SMC is mediated by gap junctions [32]. The main structures of gap junctions are connexons. Each connexon is made up of four or six connection proteins, and the Cx43 is the most important connection protein in GI tract whose expression was closely related to GI motility [40]. In many GI motility diseases, for example, diabetic gastroparesis, hypertrophic pyloric stenosis, and congenital megacolon, the expression of CX is abnormal [20].

ICC network, ENS-ICC-SMC network and gap junctions are related closely to GI motility, and there is GI motility dysfunction in MODS, but the study on changes of ICC network, ENS-ICC-SMC network, and gap junctions in MODS is still lacking.

According to the Nemeth's method [25], ICC could be detected by C-Kit immunofluorescence staining. In this study, the changes of ICC-DMP network in small intestine in MODS were investigated with c-Kit immunohistochemistry using confocal microscopy. The results showed that, in MODS group, the number of ICC was significantly decreased, the ICC network was damaged.

In this study, the changes of ultrastructures of ICC-DMP in intestinal tissues in MODS were observed using electron microscopy. The results showed that ultrastructures of ICC were damaged significantly in MODS. The ultrastructural damages to ICC may be the main cause of morphological and functional damages to ICC. The possible causes of ultrastructural damages to ICC may be gut ischemia and anoxia, the release of a large number of inflammatory mediators, and so forth [41–48]. The damages to ultrastructures of ICC were repaired by DCQD.

The results above suggest that, in MODS, the damages to ultrastructures of ICC and ICC network would affect enteric neurotransmission and the contraction or relaxation of SMC and lead to GI motility dysfunction. DCQD could effectively repair the damages and promote the recovery of GI motility.

Cholinergic neurons are the largest number of neurons in ENS [49]. Acetyl choline (AchE) is a kind of excitatory neurotransmitter released by cholinergic nerves and plays an important role in regulation of GI motility [50–52]. In addition to the sympathetic and parasympathetic nerve, there are nonadrenergic and noncholinergic nerves in intestinal tissues, for example, peptidergic nerve and nitrergic nerve. NO released by nitrergic nerve lead to relaxation of enteric smooth muscle and has inhibitory effect on GI motility. According to the Nemeth's method [25], ICC could be detected by c-Kit immunofluorescence staining, and cholinergic/nitrergic nerve could be detected by VAChT/nNOS immunofluorescence staining.

In this study, the changes of enteric nerves-ICC network in intestinal tissues in MODS were observed with double immunofluorescence staining of VAChT/nNOS and c-Kit using confocal microscopy. The results showed that the numbers of ICC and cholinergic/nitrergic nerve fibers were decreased significantly, the connections between nerves and ICC were reduced or disappeared, and nerves-ICC network integrity was damaged significantly in MODS group. The numbers of ICC and cholinergic/nitrergic nerve fibers were increased and the damages to connections were repaired in DCQD group.

In this study, the changes of ultrastructures of enteric nerves-ICC network in intestinal tissues in MODS were observed using electron microscopy. The results showed that ultrastructures of ICC and the connections between cholinergic/nitrergic nerves and ICC, ICC and ICC, and ICC and SMC were damaged significantly in MODS group. The damages were repaired in DCQD group.

The results above suggest that, in MODS, the damages to ENS-ICC-SMC network in part may be the reason of GI motility dysfunction. DCQD could effectively repair the damages and maintain integrity of the ICC network and ENS-ICC-SMC network, so it could promote the recovery of GI motility.

In this study, we observed the changes of expression of Cx43 in intestinal tissues in MODS using light microscopy and transmission electron microscopy, for qualitative, semiquantitative and quantitative analysis of Cx43. The results of immunohistochemistry showed, in MODS group, Cx43 in intestinal tissues was decreased significantly and distribution of Cx43 was not even. Gap junctions between ICC and ICC, ICC and SMC, and SMC and SMC were decreased or disappeared using transmission electron microscopy. In DCQD group, Cx43 was increased significantly and damages to gap junctions were repaired.

The results above suggest that, in MODS, decrease in numbers of gap junctions, destruction of the structure, and decline in function will affect signal transduction between ICC and ICC, ICC and SMC, and SMC and SMC. Then they will affect generation and transmission of slow wave and affect contractive function of smooth muscle, and the electrical excitation between smooth muscle cells cannot spread rapidly. These will lead to GI motility dysfunction. DCQD could effectively repair the damages to gap junctions and promote the recovery of GI motility.

Studies have shown that DCQD has anti-inflammation effect [53, 54]. The therapeutic effect of DCQD in MODS may be due to its anti-inflammation effect. But, the exact mechanism of different types of ICC in the GI motility is not fully understood, physiopathological change of ENS-ICC-SMC network in MODS is complicated, and mechanism of DCQD therapy may be multicomponent and multitarget. It remains to be further investigated.

## 5. Conclusion

The pathogenesis of GI motility dysfunction in MODS in part may be due to the damages to enteric nerves-ICC-SMC network and gap junctions. The therapeutic mechanism of DCQD in part may be that it could repair the damages and maintain the integrity of enteric nerves-ICC-SMC network.

## Figures and Tables

**Table 1:** Cx43 in small intestinal muscularis.

	Control (*n* = 30)				MODS (*n* = 30)				DCQD (*n* = 30)				*H* value	*P*
	++	+	±	−	++	+	±	−	++	+	±	−		
Visions	23	3	3	1	2	4	5	19	20	5	2	3	44.537	0.000
